# College Dispensary

**Published:** 1868

**Authors:** 


### EDITORIAL AND MISCELLANEOUS.

---

### COLLEGE DISPENSARY.

For two years, patients have been prescribed for in Atlanta Medical College, and medicine furnished and prepared for their use. This arrangement applies only to those having chronic or other diseases which admit of their coming from their homes to the College for this purpose, and who have not the means of paying the ordinary fees in private practice. It affords such persons that relief from suffering and disease they so much need, and which they could not obtain unless at a large outlay of money by the Government or City authorities in the establishment and operations of an exteusive elemosenary institution.

Under the general supervision of Dr. N. D'Alvigny, the Dispensary is made to subserve purposes of humanity, and relieve the hospital, of which he is Surgeon, from the care of hundreds which would otherwise be a tax upon it. At the same time the Faculty of the College, who make themselves responsible for the thorough investigation of, and prescription for, the cases applying, insure all the advantages to the class of students in the College, that are obtained from so large a number and extensive variety of cases.

We give below the number of cases, each, of the various diseases treated the past eight months:

| | | | |
|---|---|---|---|
| Neuralgia | 40 | Anasarca | 4 |
| Odontalgia | 18 | Ovarian Tumor | 1 |
| Amaurosis | 1 | Eczema | 4 |
| Feruncle | 1 | Vertigo | 1 |
| Hernia | 1 | Pharyngitis | 5 |
| Syphilis | 98 | Stomatitis | 2 |
| Intermittent Fever | 66 | Urticaria | 1 |
| Diarrhœa | 16 | Prolapsus Uteri | 3 |
| Influenza | 52 | Tonsilitis | 7 |
| Chorea | 2 | Uterine Engorgement | 1 |
| Indigestion | 25 | Balanitis | 1 |
| Pericarditis | 2 | Hydrothorax | 1 |
| Scabies | 41 | Necrosis | 1 |
| Ulcers | 12 | Hypochondria | 1 |
| Bronchitis | 21 | Staphyloma | 1 |
| Epilepsy | 2 | Convulsions | 2 |
| Goiter | 5 | Lumbago | 1 |
| Phlegmasia Dolens | 1 | Spinal Irritation | 3 |
| Amenorrhœa | 20 | Adenitis | 2 |
| Aneurism | 1 | Ambustio | 1 |
| Val. Disease of Heart | 2 | Glaucoma | 2 |
| Ophthalmia | 12 | Cataract | 1 |
| Rheumatism | 28 | Pneumonia | 8 |
| Leucorrhoa | 5 | Paronychia | 3 |
| Endo-metritis | 10 | Carbuncle | 1 |
| Mammary Abscess | 3 | Spleenitis | 1 |

| | | | |
|---|---|---|---|
| Aphtha | 1 | Croup | 1 |
| Gastritis | 4 | Hæmoptisis | 1 |
| Pleuritis | 4 | Abscess | 19 |
| Dysentery | 12 | Orchitis | 4 |
| Hepatitis | 5 | Ascitis | 4 |
| Hæmaturia | 1 | Laryngitis | 7 |
| Caries | 2 | Cystitis | 3 |
| Phthisis | 4 | Asthma | 2 |
| Hypertrophy of Heart | 1 | Tumors | 8 |
| Menorrhagia | 6 | Cornitis | 1 |
| Dysmenorrhœa | 11 | Icterus | 3 |
| Dentition | 4 | Nephritis | 1 |
| Remittent Fever | 5 | Tinea Capitis | 1 |
| Worms | 15 | Iritis | 6 |
| Gonorrhœa | 20 | Metritis | 18 |
| Constipation | 25 | Paralysis | 2 |
| Scrofula | 20 | Wounds | 6 |
| Cephalalgia | 13 | Ptyalism | 2 |
| Pregnancy | 6 | Menengitis | 1 |
| Debility | 14 | Ovaritis | 2 |
| Œdema | 1 | | |

From the above it will be seen that during the eight months, ending the first of May, 1868, eight hundred and ten persons were treated in the Dispensary, and that ninety-three diseases were presented during the time. It appears that syphilis, intermittent fever, influenza, scabies, neuralgia, and rheumatism, in the order we now mention them, were the prevailing disorders. The predominance of scrofula over phthisis is somewhat remarkable; but when the fact is taken into consideration that a large majority of the subjects were negroes, and that the colored race are, in this latitude, more subject to the development of the former than the latter, from the depressing influences of the want of substantial comforts, the explanation is easy. The number affected with scabies, or itch, is not to be wondered at, for the reason that the want of cleanliness and the crowding together of large families of children tend to engender and perpetuate its existence.

The number affected with uterine disease of various kinds is fifty-eight. Of these, five with lucorrhœa and six pregnancy, the special difficulty requiring treatment is not specified, but a sufficient number have their disease definitely indicated to prove conclusively that uterine affections are not confined to ease and luxury.

